# Aneurismal Dilatation of the Posterior Auris and Temporal Arteries
*Edinburgh Med. and Surg. Journal, Jan. 1, 1829.


**Published:** 1829-04-01

**Authors:** 


XXIII.

### Aneurismal Dilatation of the Posterior Auris and Temporal Arteries.* By James Syme, Esq.

We are led to notice this case for two good reasons; first, because it is an example of a disease which surgeons are only beginning to understand; and secondly, because it illustrates those principles of treatment on which we have occasionally dwelt with considerable earnestness. John Bell is the only writer who has published any more than an insulated case of the disease in question, and he, as is very well known, has given it the name of "aneurism from anastomosis." Whether that term be generally proper or improper we need not stop to inquire; our business at present being only with those pulsating tumours which appear on the side of the head. To these particular cases there cannot be a doubt that both the name and patho-

---

* Edinburgh Med. and Surg. Journal, Jan. 1, 1829.

logical description furnished by Mr. John Bell do not, with strict propriety, apply, the disease consisting not in a "congeries of small and active arteries, absorbing veins, and intermediate cells," but in mere dilatation, with thinned parietes, of one or more of those arterial branches which supply the scalp. Four well-marked cases are now recorded, the first of which occurred to Pelletan, the second to Mr. Wardrop at the "Hospital of Surgery," the third to Dr. Maclachlan at Glasgow, and the fourth, which we are now to notice, to Mr. James Syme at Edinburgh. The three former cases had all ended fatally, the latter has been successfully treated.

In Pelletan's case, the disease being seated in the temporal and occipital arteries, pressure was attempted to be applied over the former, but it caused such pain that the surgeon was forced to desist. M. Pelletan then tried to put a ligature upon the vessel, which he did, though at the same time he managed to transfix its coats. The pulsation in the tumour stopped, but according to Boyer, an unsuccessful attempt was also made to secure the occipital artery by diving through the scalp with a sharp needle. This M. Pelletan says nothing about, nor yet of the pain and repeated hæmorrhage which followed, and are said by M. Boyer to have been the causes of the patient's death. M. Pelletan, indeed, with a candour which would amply qualify him to cut a figure even in these flagitious days, merely states that she died of indigestion!

Mr. Wardrop's case, which excited a considerable sensation at the time, was one of those famous Panton-Square Reports, which stuck so many laurels round the wig of the ingenuous reporter! In the "invaluable" account of the case, it was asserted that the trunk, the anterior, and the posterior branches of the temporal artery were affected, whilst the posterior auris, the occipital, and a branch of the opposite temporal were "wonderfully enlarged and communicated abruptly with the disease." The cranium below "was found (at Panton Square) to have undergone a remarkable process of absorption, particularly at those points where the tumour had attained its greatest bulk. It must at many places have become exceedingly thin." Mr. Wardrop tied the common carotid, and wounded or tied the internal jugular vein, with the effect not of curing the disease, but of causing the loss of one eye. The patient died two months afterwards in the Middlesex Hospital, and the dried preparation of the parts is now in the Anatomical Museum of Great Windmill Street. The trunk and the anterior branches of the temporal artery were unaffected, but the posterior divided into two, one of which perforated the tendon of the occipito-frontalis, and then became dilated and tortuous, and coiled upon itself, like a varicose vein. The "posterior auris, the occipital, and the branch of the opposite temporal," had been "wonderfully enlarged," only in the fertile fancy of the Panton Square reporter, for, on dissection, no such astounding appearances were found. The "remarkable process of absorption" in the cranium and "exceeding thinness," were also nowhere to be found, except in the Lancet report. The common carotid artery, from the site of the ligature up to its bifurcation, and the *jugular vein* for an equal space, were converted into mere cellular chords—there was pus at the basis cranii and around the commissura tractuum opticorum, which continued between the pia mater and arachnoid, down the whole length of the spinal chord.

We mention these circumstances, although we have detailed them more fully in a former number of the Journal,* first, that the present account of all that is known on the disease may not be incomplete; and secondly, for the especial edification of Mr. Syme. We are neither by habit nor inclination hypercritics, but we cannot avoid remarking on the want of fairness or the want of information displayed by Mr. Syme in the mention he has made of this case. He gives the ridiculous statements of the Lancet, and says not a word of the actual condition of the parts as shewn by *dissection* and detailed in this Journal, as well as in the Medical Gazette.† Is Mr. Syme one of those gentlemen who read only the Invaluable? If so, he need not be surprised nor offended at experiencing a rub now and then in this his mortal literary career; if not, he should show it by recording something else besides the trash

* No. XVI. page 500. April, 1828.

† No. IX. page 260.

to be found in that journal. No wonder, forsooth, that medicine abounds in "false facts," when one man publishes, with his eyes open, the absurd or erroneous reports of another.* The next time Mr. Syme is at the pains of quoting cases from the Lancet, perhaps it would be as well to ascertain whether or not they have been proved to be incorrect. The hospital reports in that publication are notoriously of so doubtful a character in point of common accuracy, that no one, with a grain of discretion, would commit himself by attaching any weight to them.

The third case of this very curiors disease is that which was published by Dr. Maclachlan in the second number of the Glasgow Journal, and fully detailed by ourselves at the time.† The temporal, posterior auris, occipital, and their principal branches were affected, being thin in their parietes, dilated, and convoluted on themselves, like clusters of varicose veins. Pressure had been ineffectually employed, when Dr. Maclachlan tied the temporal, and, not exactly liking the state of this vessel, secured the carotid next day. Four days afterwards the patient died, and, on dissection, pus was found in the anterior mediastinum, with a pint of greyish muco-purulent matter in the right cavity of the pleura. The ligature of the common carotid had little or no effect on the disease, and the branches of the artery were found to be perfectly pervious.

That this affection differs from common aneurism, especially from "false" aneurism, where the internal coat of the artery is ruptured, is obvious enough. It has long been known that the blood is more apt to coagulate when lying in an aneurismal sac, especially a sac formed by rupture of the internal coats of the artery, than when merely contained in a *dilated* vessel. On this important principle has depended, in great measure, the success of the Hunterian operation for aneurism, viz. tying the artery at a more or less considerable distance from the disease. Fascinated, however, by this triumphant discovery, surgeons have run riot in its application, and used it and abused it to a ridiculous degree. Their heads have been so wild, and their hands so bold, that they fancied they had nothing to do but tie a great blood-vessel in order to arrest a hæmorrhage or cure an arterial dilatation even at the most extravagant distance from the spot where they performed this operation! In hæmorrhage they have already discovered their mistake; in these cases of arterial dilatation we fancy they will quickly do so. Can any thing be more idle than the notion of curing an affection of the branches of the temporal and occipital arteries, like that we have described above, by tying the carotid? Our sentiments on this point were long ago made known.

"You tie the carotid, you expect to obliterate its dilated temporal branch, and why? Is it because a similar operation on the femoral artery will cure a popliteal aneurism? The analogy is not a fair one. Between the point of the ligature and the sac, there come off, in the femoral, no branches of any consequence. One or two of the articular arteries may arise just above the sac, but they are small, and, at any rate, anastomose with the tibials below, so as not to interrupt the remora in the sac itself. The new circulation is carried on principally by the branches of the profunda and common femoral, (we mean its long external descending ramus,) and *not by the vessels of the artery tied.*

"With the carotid it is exactly the reverse. First, there is the division into internal and external, and, prior to this last terminating in the internal maxillary and temporal, there are *eight branches given off*, all of them having communications more or less free with each other. This being the case, we put it boldly to any man of common sense, whether he can reasonably expect to obliterate an enlargement of the *terminal* branch of a vessel, whilst a full and free circulation *must* necessarily be kept up between that enlargement and the ligature. The consequence of tying the common carotid is simply this, that the vessel is obliterated nearly as high as its division into the external and internal, but not one jot higher, because, above this, the circulation is brought *into* these vessels by their anastomosis with the inferior thyroid of the same side, and the thyroid and other

* See the *fictitious* lectures and communications of Mr. Liston in the Lancet!

† No. XVI. New Series, 497.

branches of the opposite, as well as by the free inosculations of the internal carotid. In fact, the man who imagines he can plug up the temporal artery by an operation on the common carotid, might just as fondly attempt to obliterate the vessel of the great toe by tying the artery at the groin!"

These were our sentiments expressed in February, 1828, and these are our sentiments in February, 1829. From the disease consisting in *mere dilatation* of one or more arteries of the scalp, together with their excessive freedom of anastomosis, one with the other, we contended then and contend now that its characters differ most essentially from those of aneurism, that it will not admit of the same treatment, that it cannot be cured in the same way. We have said, it was absurd to tie the carotid, but in practice, alas! the operation has been more than absurd — it has been fatal. Securing the temporal artery, particularly when the branches of that vessel only were affected, might appear a more mild and more rational plan than tying the carotid trunk, with all the intermediate inosculating branches remaining. In that experimentum crucis—practice, the ligature of the temporal has proved indeed more mild, but not a whit more powerful in checking the disease. In each of the three cases above detailed it was had recourse to without effect, a circumstance very easily explained by the freedom of anastomosis subsisting betwixt the artery diseased and its fellow of the opposite side, or the neighbouring branches of other vessels. It comes then to this, that your scientific, Hunterian operation will never do for such a disease. You must not be over nice, you must cut into the tumour, or cut it out, or at all events you must do that which shall effect a radical and substantial cure.

In our article on the subject in the 16th Number of this Journal, to which we have already alluded, we recommenmended *circumvallating* the tumour by an incision, or at any rate, tying the branches going *to* it and *from* it. The fact, however, is, at least so it appears to us, that the *mode* of operation must vary according to circumstances, although the principle will still be the same in all. If, as in Dr. Maclachlan's case, the disease be not confined to a single branch, but extend to several and occupy, in different ramifications, nearly the half of the head, it is obvious that excision or incision, at all events the former, would be out of the question. In such a case, would it not be right to tie the vessel going to and from *each* varicose enlargement, either at one operation, or, which perhaps is better, at intervals? Such an operation would not be difficult, would, cæteris paribus, not be dangerous, and would cure the disease. Nothing short of it, in our opinion, would be effectual, and nothing, that we know of, which promises to be effectual, would be less severe.

When, however, the tumour is confined to a single vessel, say the temporal, is not very large, and is circumscribed, some other, and in this instance probably preferable operations, present themselves. If the tumour were not very tortuous, the old operation for aneurism might be resorted to, namely, cutting into it, and tying the vessel passing to and from the disease. If, however, the dilated vessel should have grown very tortuous and coiled upon itself, as after a certain lapse of time it does, this plan of proceeding would obviously not be appropriate, and perhaps it would be better to adopt that first recommended by Mr. Mayo,—excision. Unless the tumour be very large, and implicate several vessels at once, we know of no particular objection to this operation. It was perfectly successful after the ligature of the temporal artery had failed, in the case of Mr. Syme, which forms the subject of the present paper, and of which we shall now subjoin the particulars. We have thought it right for the full understanding of the case, or at all events, of our own ideas of the disease, to make the preceding observations. We are not, in general, given to indulge in critical remarks, and therefore we hope to be excused the more readily, when we do depart from rigid analysis.*

The patient was a Mrs. T., aged about 50, by whom Mr. Syme was consulted,

---

* We hear that Mr. Brodie has successfully treated a case of the disease by a modification of the ligature applied to the base of the tumour, after the artery feeding it had been ineffectually tied. The case fully supports the *principle* we have been advocating.

in the middle of last July, on account of a tumour, the size of a large gooseberry, situated behind the right ear, over the mastoid process. At first sight it looked like a common encysted tumour, but on using pressure it was emptied, and immediately filled again when the pressure was removed. During the time that the tumour was filling, a jet of blood could be felt issuing from the bottom of the tumour, and a whizzing noise was heard by the patient most distinctly. The posterior auris was felt enlarged and pulsating with violence below, and when it was compressed the tumour became flaccid. The lady complained of pain and noise in the swelling, the latter being often so distracting as to deprive her of her sleep. The disease had appeared ten years previously, after an accouchement, and had latterly increased with greater rapidity. Pressure had been tried without advantage.

Believing the disease to be aneurism of the posterior auris, Mr. S. in conjunction with Dr. Ballingall, proceeded to tie that artery next day. However, on shaving off the hair they found to their concern that the disease extended in the course of the artery, and finally, that not only all its branches were dilated, but also the posterior and middle branches of the temporal, all of which were throbbing obviously, though not very strongly. A plate is appended shewing the disease, but it seems pretty clear that only the posterior auris can be said to be affected with the disease, the branches of the temporal being rather enlarged by anastomosing with the former dilated vessel, than actually implicated in the same morbid state.

"We now *thought it would be necessary to tie the carotid*, but before doing so fortunately discovered that when the posterior auris was compressed the dilatation disappeared; we therefore proceeded to execute our original intention. I exposed the vessel a little below where it entered the tumour, which was not very easy, as its course was perpendicular to the surface, and tied it with a single silk ligature. It was about the size of the radial and proportionally very thin in its coats. When the ligature was drawn the tumour became flaccid and the dilated vessels disappeared. The edges of the wound were kept together by two stitches, and a compress moistened with acetate of lead water was applied to the rest of the head.

"Every thing went on well for a week, excepting a slight attack of erysipelas, which was to be expected, as the patient informed me she frequently suffered from bilious attacks.

"On the eighth day after the operation, while I happened to press on the tumour, a slender stream of arterial blood trickled away from the side of the ligature. As it soon ceased I merely applied a compress over the wound. The hemorrhage recurred twice or thrice in the twenty-four hours on the following days,—but as it never exceeded an ounce or two I concluded that it came from the vessel above the ligature, and therefore contented myself with using superficial pressure, not in the expectation of arresting the discharge of blood,* but in the fear of disturbing, by more efficient measures, the process of obliteration going on below the ligature, which would have been attended with more serious consequences.

"On the twelfth day, conceiving that the ligature must have done its duty, I examined the wound, and found in the seat of the ligature a small pulsating bag, from a crevice in the centre of which the blood escaped. Having detached with my nail this little false aneurism, and along with it the ligature which was inclosed, I ascertained that the hemorrhage did proceed from the orifice of the vessel next the tumour. I then applied some small pieces of amadou supported by a graduated compress.

"Every thing went on well afterwards. I dressed the wound at the end of three days, when it was suppurating most sa-

"* It is highly important for surgeons to recollect that pressure is of little avail in the stopping of hemorrhage unless it is applied directly to the bleeding vessel. If this truth were kept in mind we should not so often hear of the humeral artery being tied, since I will venture to affirm, that there is no bleeding from injury of the hand, and I will add of the foot, which cannot be commanded by local pressure. But the pressure must be applied to the bottom of the wound, and if the orifice is not wide enough to admit of this it ought to be dilated."

tisfactorily, and in the course of a short time it cicatrized.

"For some weeks after the operation the tumour remained small and flaccid, but when the patient resumed her ordinary diet and exercise, it began to resume its former condition. It was moderately tense; and though no throbbing in it could be felt by the finger, Mrs. T. complained of the noise and pain which had distressed her previously, in a degree comparatively slight, but sufficient to disturb her repose. No appearance of the varicose dilatation of the artery could be perceived.

"Finding that the uneasy symptoms continued to increase, and being anxious to take advantage of the command which had been obtained for the present over the disease by obstructing the principal supply of blood, I determined to take an effectual step for the patient's relief.

"On the 29th of October, assisted by Professor Ballingall, I cut directly through the long direction of the tumour, which then shewed itself to be composed of large irregular cells, invested by a firm capsule. While Dr. B. compressed above and below the tumour, I dissected it out, and then attempted to tie the vessels, but finding this very difficult, I adopted the suggestion of Dr. B. and included them in ligatures by means of a small curved needle. The ligature being drawn, the hemorrhage ceased. I then filled the wound with dry caddis, and applied a firm bandage about the head. The patient did not experience the smallest inconvenience from this operation, excepting the pain immediately attending it. The ligatures separated in about a fortnight, and the wound is now completely healed.

"I am induced to publish this case, 1. Because it throws light on the nature of aneurism by anastomosis. Most surgeons have followed John Bell in thinking that this disease consists of a morbid cellular structure, through which the blood passes in its course from the arteries into the veins. I have long been one of those who maintain that the apparent cells are really sections of enlarged vessels, a good illustration of which opinion is the appearance presented by cutting across the injected spermatic cord of a ram's testicle—or a comparison of the glans penis in man or any other animal, where it seems to consist of cells, with that of the ram or fallow-deer, where it is most distinctly formed by convoluted vessels. In the cases of Pelletan, Wardrop, and Maclachlan, this structure is more readily traced; and in the case I have related it exists so obviously as to admit of no question.

"2. Because it shows that the thin dilated arteries are capable of the obliterating process."

This case fully illustrates the remarks we have made on the disease, and requires no further comment. We think, however, that if Mr. Syme and Dr. Ballingall reflect for a minute on the nature of the disease, and the history of the cases which have now been published, they will alter their opinion as to the necessity, or even the propriety of tying the carotid artery. The case of tumour within the orbit, for which Mr. Travers performed the above operation, has been instanced as a case in point. If any one will take the trouble to read the details of that case, they will find that nature did more towards effecting the cure than the surgeon. The disease was actually returning, and we stake our existence would have returned, had it not been for the fortunate occurrence of that uterine hæmorrhage, which helped the surgeon, saved the patient, and established a dangerous, nay, fatal precedent!

